# Frequency of Detection and Phylogenetic Analysis of *Porcine circovirus* *3* (PCV-3) in Healthy Primiparous and Multiparous Sows and Their Mummified Fetuses and Stillborn

**DOI:** 10.3390/pathogens9070533

**Published:** 2020-07-02

**Authors:** Viviane Saporiti, Susanna Martorell, Taís F. Cruz, Francini Klaumann, Florencia Correa-Fiz, Mònica Balasch, Marina Sibila, Joaquim Segalés

**Affiliations:** 1 IRTA, Centre de Recerca en Sanitat Animal (CReSA, IRTA-UAB), Campus de la Universitat Autònoma de Barcelona, 08193 Bellaterra, Spain; viviane.saporiti@irta.cat (V.S.); tfcruz@yahoo.com.br (T.F.C.); franciklaumann@gmail.com (F.K.); flor.correa@irta.cat (F.C.-F.); marina.sibila@irta.cat (M.S.); 2 OIE Collaborating Centre for the Research and Control of Emerging and Re-emerging Swine Diseases in Europe (IRTA-CReSA), 08193 Bellaterra, Barcelona, Spain; 3 Zoetis Manufacturing & Research Spain S.L., Ctra. Camprodon s/n, La Riba, 17813 Vall de Bianya (Girona), Spain; susanna.martorell@zoetis.com (S.M.); monica.balasch@zoetis.com (M.B.); 4 Department of Immunology and Microbiology, Institute of Biosciences, São Paulo State University (Unesp), Botucatu 18618-970, Brazil; 5 Departament de Sanitat i Anatomia Animals, Universitat Autònoma de Barcelona (UAB), 08193 Bellaterra, Barcelona, Spain; 6 UAB, Centre de Recerca en Sanitat Animal (CReSA, IRTA-UAB), Campus de la Universitat Autònoma de Barcelona, 08193 Bellaterra, Spain

**Keywords:** mummified fetus, stillborn, sow, Porcine circovirus 3, vertical transmission, intra-uterine infection

## Abstract

Porcine circovirus 3 (PCV-3) has been suggested as a putative causal agent of swine reproductive disease. A number of different studies have pointed out this association, but there is still a lack of information regarding the normal rates of PCV-3 infection in farms with normal reproductive parameters. The objective of the present study was to assess the frequency of PCV-3 detection in primiparous and multiparous sows and in tissues from their respective fetuses from farms with average reproductive parameters. Sera from 57 primiparous and 64 multiparous sows from 3 different farms were collected at two time points. Brain and lung tissues from 49 mummies and 206 stillborn were collected at farrowing. Samples were tested by PCR, and when positive, quantified by quantitative PCR. Thirty-nine complete genomes were obtained and phylogenetically analyzed. All sera from multiparous sows were negative, while 19/57 (33.3%) primiparous sows were PCV-3 PCR positive. From the 255 tested fetuses, 86 (33.7%) had at least one tissue positive to PCV-3. The frequency of detection in fetuses from primiparous sows (73/91, 80.2%) was significantly higher than those from multiparous ones (13/164, 7.9%). It can be concluded that PCV-3 is able to cause intrauterine infections in absence of overt reproductive disorders.

## 1. Introduction

Porcine circoviruses (PCVs) are small DNA viruses and have four representatives, PCV-1, PCV-2, PCV-3, and tentatively, PCV-4. PCV-1 is known as non-pathogenic for pigs [1], while PCV-2 has been associated with several conditions known as porcine circovirus diseases (PCVDs) [2]. PCVDs include PCV-2 systemic disease (PCV-2-SD), PCV-2 reproductive disease (PCV-2-RD), porcine dermatitis and nephropathy syndrome (PDNS), and PCV-2 subclinical infection (PCV-2-SI) [2]. The PCV-2-SI is probably the cause of the greatest economical losses for the pig industry [3]. In 2015, PCV-3 was firstly described in sows displaying reproductive failure and PDNS [4], as well as in pigs with multisystemic inflammation [5]. Since then, many other descriptions of the virus presence came up from pigs displaying a number of different diseases and even in healthy animals [6,7,8]. PCV-4 is the newest tentative member of the *Circoviridae* family and was described in pigs displaying respiratory and digestive clinical signs as well as PDNS [9]. It is already known that PCV-1, PCV-2, and PCV-3 are ubiquitous pathogens [1,2,7], while PCV-4 has been only detected in China so far [9,10].

The PCV-3 genome was initially found in cases of reproductive disorders, specifically in mummified fetuses and abortions [4]. Although PCV-3 pathogenesis is poorly known, a high number of reports have pointed out a potential and causality association of PCV-3 with reproductive disease based on virus detection and clinical signs in the absence of other pathogens [4,5,11,12,13,14,15]. This putative association is also supported by a newly released study where the virus was successfully isolated from cases of reproductive losses [16].

The PCV-3 genome is composed of 1999–2001 nt [17,18] with two well-characterized open reading frames (ORFs), ORF1 encoding the replicase protein (Rep) and ORF2 encoding the capsid protein (Cap). ORF1 and ORF2 are located in positive and negative strands, respectively [4,5] (Phan et al., 2016; Palinski et al., 2017). Although being from the same family, PCV-2 and PCV-3 are far different in terms of amino acid (aa) homology, sharing only 48% of identity in the *rep* protein [5] (Phan et al., 2016) and between 26% and 37% in the *cap* protein [4,5] (Phan et al., 2016; Palinski et al., 2017). Despite PCV-3 available sequences sharing high similarity among them, different classification systems based on aa marker positions have divided PCV-3 into two (PCV-3a and PCV-3b) [17,19] or three (PCV-3a, PCV-3b, and PCV-3c) [20] main groups. However, a recent study highlighted several exceptions for the mentioned marker positions and proposed a definition based on only one PCV-3 genotype to date, the PCV-3a [21].

Considering the potential association of PCV-3 with reproductive cases, the objective of this study was to assess the frequency of detection of PCV-3 and phylogenetically analyze the virus in serum samples from primiparous and multiparous sows from farms without reproductive problems, as well as in tissues from the respective mummified or stillborn piglets. This study was performed to establish the “PCV-3 infection background” in normally performing farms.

## 2. Results

### 2.1. PCV-3 Detection and Virus Quantification

From the 121 sows included in the study, 19 (15.7%) had at least one PCV-3 PCR-positive serum sample. All these positive samples corresponded to primiparous sows (19 out of 57, 33.3%) from the three different farms (Table 1). From these, one was collected at sampling point 1 (S1) and 18 were collected at sampling point 2 (S2) (*P*-value = 0.001); the positive sample at S1 was negative at S2. The difference in frequency of positivity between S1 and S2 was highly significant (*P*-value < 0.0001). However, the frequency of PCV-3 PCR positivity in primiparous sows between the three farms was not significantly different. All sera from multiparous sows were negative at both sampling times. 

The viral load of the positive sera from primiparous sows ranged from 0.47 to 2.30 log_10_ copies/µL, with the exception of one sample with a viral load below the quantification limit of the technique (one copy of DNA/µL). 

Globally, the number of PCV-3 PCR positive fetal samples from primiparous sows (73 out of 91, 80.2%) was significantly higher (*P*-value < 0.0001) than the one from multiparous ones (13 out of 164, 7.9%). These statistically significant differences were also observed in either farm A or C, when individually analyzed (Table 2). Additionally, the median PCV-3 load found in fetuses’ tissues from multiparous sows (1.16 log_10_ copies/µL) was significantly lower (*P*-value < 0.0001) when compared to the viral load in fetuses’ tissues from primiparous sows (3.57 log_10_ copies/µL). Moreover, the viral loads were similar in positive fetuses from the same litter; however, among mummies and stillborn from the same sow, mummies tend to have higher viral load. 

The number of mummies with at least one PCV-3 PCR positive sample (27 out of 49, 55.1%) was significantly higher (*P*-value = 0.0008) than that of the stillborn (59 out of 206, 28.6%) (Table 2). The total number of PCV-3 PCR-positive mummies compared to stillborn was statistically significant in farms A and C (*P*-value = 0.0005 and *P*-value = 0.0467, respectively). No statistically significant differences in the median PCV-3 viral load detected in the tissues from mummies (3.56 log_10_ copies/µL) and stillborn (2.93 log_10_ copies/µL) were found.

Twenty-one out of the 27 (77.8%) and 40 out of the 59 (67.8%) PCR-positive mummies and stillborn, respectively, were positive in both tissues analyzed (lungs and brain). The frequency of PCV-3 genome detection in fetal lungs (81/255, 31.8%) was numerically higher when compared to that of the brain (66/255, 25.9%), but these differences were not significant (Table 3). The percentage of detection in brain and in lung was significantly higher in mummies than in stillborn in farms A and C (*P*-value < 0.05) (Table 3). The PCV-3 load ranged from 0.38 to 7.16 log_10_ copies/µL in brain and from 0.15 to 7.84 log_10_ copies/µL in lungs. There were three samples from brain and three from lungs, from different animals, with viral loads below the quantification limit of the qPCR. No statistical differences in viral load were found between lung and brain tissues in fetuses neither from primiparous nor from multiparous sows.

### 2.2. PCV-3 Phylogenetic Analysis

Forty-two PCR positive samples were selected for the phylogenetic analysis, being 8 serum samples from primiparous sows, 16 tissues from mummified fetuses (8 brains and 8 lungs), and 18 tissues from stillborn (8 brains and 10 lungs). It was possible to achieve 39 complete genome sequences out of the 42 selected samples. The sequence from the remaining 3 samples (brain and lung from one stillborn and the lung from another one) showed bad quality. The nucleotide identity of these 39 samples ranged from 99.2% to 100%. When possible, PCV-3 sequences from fetuses of the same litter, as well as the respective sow, were compared. This comparison was done in four cases, and the identity of the viral sequences obtained ranged from 99.30% to 100%. Moreover, sequences from fetuses of the same litter of three other cases were compared showing an identity between 99.7% and 100%.

**Figure 1:**
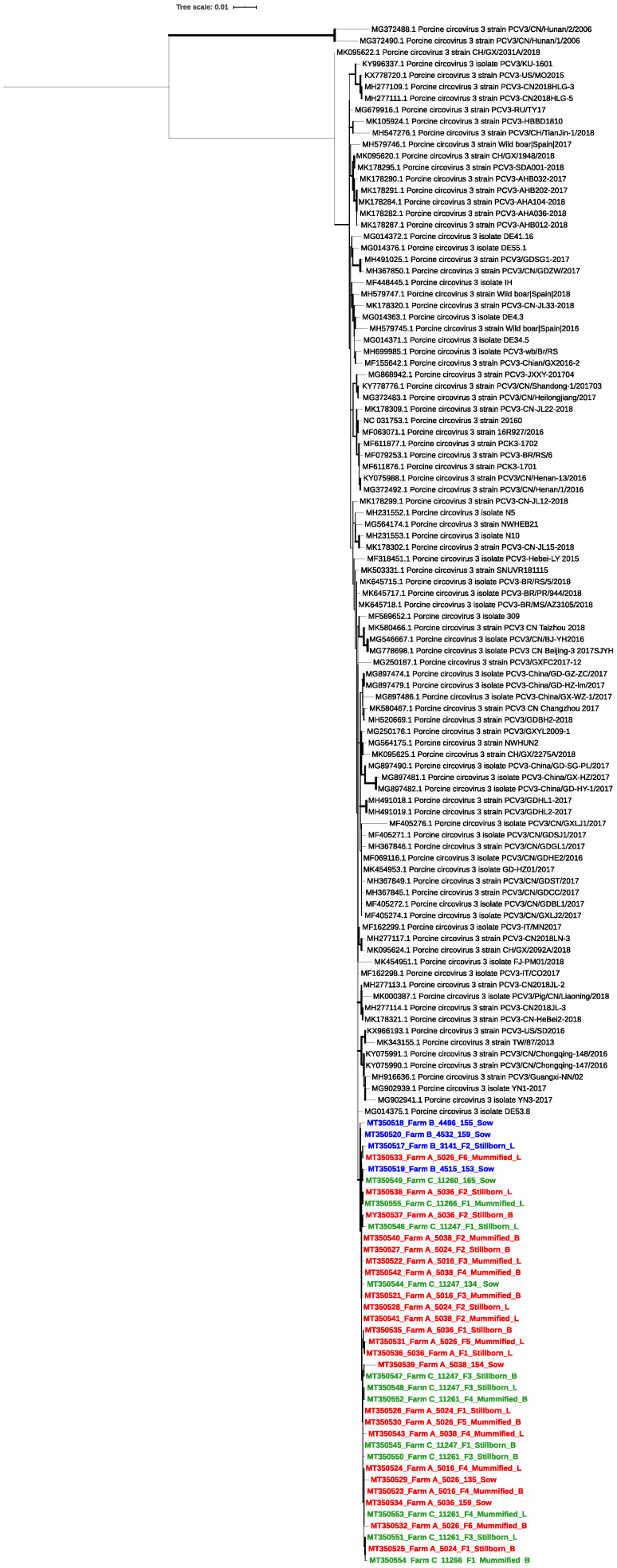
Phylogenetic analysis of PCV-3 complete genome sequences. The tree was constructed for the full genome sequences obtained herein, and the PCV-3 references sequences included in Franzo et al., 2020. The best substitution model with the highest Bayesian Information Criterion (BIC) score was used with 1000 bootstraps (Hasegawa–Kishino–Yano with Gamma distribution). The width of the branches is proportional to bootstrap *P*-values, and the scale bar indicates nucleotide substitutions per site. The sequences obtained in this study were labeled with GenBank ID followed by farm identification, sow number and sample number; F, for fetus samples; B, for brain or L for lung tissue. Samples were colored by farm (farm A in red, farm B in blue and farm C in green).

The phylogenetic analyses performed with the complete PCV-3 genome sequences showed two main clusters (Figure 1). All sequences analyzed herein belonged to Cluster 1, together with the reference sequences previously genotyped as PCV-3a [21]. Similar results were found when only the cap region was analyzed (Figure S1). The overall nucleotide identity with the PCV-3a reference sequences used for the complete genome phylogenetic tree was 97.75% to 100%. When the aa sequence inferred from the ORF2 gene was phylogenetically analyzed (Figure S2), all samples were also classified as PCV-3a [21], although with a different pattern of distribution among the reference sequences used. The aa identity of the inferred ORF2 protein was high, ranging from 98.9% to 100% among all the sequences obtained herein, supporting the fact that they all belong to the same cluster.

## 3. Discussion

The first description of PCV-3 infection was linked with reproductive losses with high viral loads in aborted fetuses [4]. Since then, this potential association has been reported in different countries. In China, a study found a higher frequency of the PCV-3 genome in sera from sows with a reproductive failure history (39/85, 45.9%) compared to healthy ones (23/105, 21.9%) [15]. In a study from Brazil, PCV-3 viral DNA was found in pooled sera from sows delivering stillbirths, while it was absent in pool sera from sows with no stillbirth delivered [14]. In one study from South Korea, the virus was suggested as the potential cause of an increased abortion and death rates of suckling piglets, since they could not demonstrate evidence of another pathogen in the tested animals [13]. A Hungarian newly populated farm experienced an increase of abortions and acute losses of neonatal piglets from the primiparous sows as well as an increase of stillborn and mummified fetuses compared to previous cycles; PCV-3 was the only pathogen found in these cases [12]. However, the most unequivocal association to date of PCV-3 association with reproductive disease has been reported from the USA [22]. The authors found the presence of PCV-3 nucleic acids by means of in situ hybridization within the lesions of mummified and stillborn fetuses, especially from low-parity sows. Specifically, the PCV-3 genome was found in the smooth muscular cells of arteries of both the heart and kidney and in inflammatory cells in the heart. 

However, taking into account that PCV-3 is a ubiquitous virus worldwide [7], the mere detection of PCV-3 is not a clue to establish a potential association with disease. Therefore, in such scenarios, it is important to elucidate to which extent and in which frequency PCV-3 does circulate in normally performing farms. In fact, most of these studies reporting an association between PCV-3 and reproductive losses lack proper negative controls with standard reproductive parameters [12,13,16,22]. Therefore, the aim of the present study was to evaluate the frequency of PCV-3 in samples from farms with good reproductive parameters. It is noteworthy that the sampled farms had the reproductive parameters within the Catalan and Spanish averages in regard to stillbirths (BDPork, Available at: http://bdporc.irta.es/informes/PartPublica/Datos%20publicos%20Anyo%202016.htm, accessed on 1 July 2020). The fact that the farms had good reproductive parameters did not completely exclude the possibility of unnoticed reproductive problems in some sows (apparently primiparous ones) that may have caused few stillbirth and mummifications.

In the present study, PCV-3 DNA was only detected in sera from primiparous sows, mainly close to farrowing time. These results are in accordance with a study from Thailand that showed a higher viral load of PCV-3 in sera from low-parity sows when compared to older parity ones [23]. These results were further confirmed by another study from the same research group, where 71% of the analyzed primiparous sows were positive to PCV-3 through the colostrum, while multiparous dams showed a shedding frequency of 33–43% [24]. Interestingly, in the last study, the viremic and non-viremic sows did not show a significant different rate of total born or born-alive piglets [24], which is also in accordance with the present work where despite the presence of the virus in the herd, no reproductive losses were observed. Specifically, the mean of total piglets born per litter in farms A, B, and C were 14.39, 14.15, and 14.79, respectively.

A wide range of PCV-3 loads was found in positive fetuses in the current work, being the highest loads similar to the ones found in studies in which the presence of the virus in fetuses was attributed to reproductive losses, as well as associated to lesions [4,22,25]. In the present study, primiparous sows had a significantly higher number of PCV-3-infected fetuses with higher PCV-3 loads compared to those coming from multiparous sows. These findings may suggest that multiparous sows may have previously developed immunity that is able to prevent PCV-3 infection in their litters. In contrast, it is hypothesized that primiparous sows started gestation immunologically naïve against PCV-3, and the potential lack of immunity to the virus may have favored viral circulation in the herd and eventual transplacental transmission [26]. This situation would resemble the one observed in PCV-2, as piglets from primiparous sows are usually more susceptible to PCV-2 and PRRSV co-infection than piglets from multiparous sows [27].

The significantly higher PCV-3 detection rate in mummified than in stillborn fetuses found in the present study is also in agreement with the results obtained by Dal Santo et al. (2020), where almost 97% of the tested mummies were PCR-positive to the virus in commercial farms from Brazil [11]. However, in this latter study, most of the infected mummies came from farms experiencing reproductive losses; moreover, the fetuses also had co-infection close to 93% with other pathogens such as *Porcine parvovirus*, PCV-2, or *Leptospira* spp. The presence of co-infecting pathogens would also explain the reproductive losses [11], thus compromising the putative association with PCV-3 in the absence of alternative diagnostic methods [22]. An Italian study from farms experiencing reproductive failure in different stages of pregnancy demonstrated a high viral load of PCV-3 in tissues from stillborn and from aborted fetuses, while the most common pathogens that can cause reproductive disease were absent [25]. Similarly, mirroring with PCV-2, it is known that vertical transmission can happen at various stages of pregnancy, being able to cause reproduction losses as well as asymptomatic outcomes, depending on the timing of the virus infection and the degree of viral replication [28]. Based on obtained results here, it may happen that PCV-3 infection mainly occurred earlier in the gestation, which would explain the higher percentage of infected mummified fetuses in comparison to stillborn.

Therefore, it is important to use additional diagnostic methods such as in situ hybridization (ISH) in order to confirm the involvement of PCV-3 in the fetal lesions. Arruda et al. (2019) found messenger RNA matching with histological findings of multisystemic inflammation in a number of different tissues, including lung and brain. These features suggest that the virus might be replicating in these tissues, thus being the most probable cause of the disease. On the contrary, Faccini et al. (2017) observed that lungs from PCV-3 PCR-positive aborted fetuses did not show histological lesions despite having found high amounts of this virus in pools of tissues. Unfortunately, due the lack of available fixed tissue in the present study, histopathological evaluation and ISH were not able to be performed. 

The phylogenetic analysis of the herein obtained sequences showed an extremely high percentage of nucleotide identity, as also observed in many other studies [17,21,22,29,30]. The nucleotide identity was slightly higher when analyzing the virus found in fetuses from the same litter as well as from the respective sows, which further suggest the vertical transmission of the virus. 

Through the phylogenetic analysis of the complete genome and the translated aa sequence from the ORF2 gene, it was possible to classify the 39 sequences recovered herein as PCV-3a [21]. All 39 samples clustered together in a different branch from the available reference sequences when analyzed either the complete genome or the cap region (ORF2) tree, showing a highly similarity between them. However, when the translated Cap sequence was evaluated, some samples clustered separately, suggesting that some of the mutations found within this region were non-synonymous, leading to different changes in the aa tree.

The present study demonstrated the presence of the PCV-3 genome in mummies and stillborn fetuses, supporting PCV-3’s ability to cause intrauterine infections, even in farms with standard reproductive parameters. Moreover, a higher frequency of infection was found in primiparous sows and in mummified fetuses compared to multiparous dams and stillborn piglets, respectively. This study will help establish the ‘infection background’ of PCV-3 in standard farms without overt reproductive disorders. Although these results reinforce the vertical transmission of PCV-3, it is already too early to speculate about the importance of these findings, and further investigations are needed to ascertain the pathogenesis of PCV-3 infection.

## 4. Materials and Methods 

### 4.1. Samples

Sera from 121 sows belonging to 3 different farms (A, *n* = 44; B, *n* = 37; and C, *n* = 40) were collected at two time points; the first one (S1) close or at pre-mating and the second sampling (S2) close or at farrowing time. These farms showed good reproductive parameters as farms A, B, and C presented means of 0.38, 0.29, and 0.56 mummies out of 14.39, 14.15, and 14.79 mean of piglets born per litter, respectively. These values are considered even lower than the average of the mummies/stillborn of Spanish pig farms included in the BDPorc database (http://www.bdporc.irta.es/index.jsp). All farms were conventional ones, seropositive against porcine reproductive and respiratory syndrome virus (PRRSV), porcine parvovirus (PPV), and PCV-2, but negative to Aujeszky’s disease virus (ADV). The normal vaccination schedule included PRRSV and PPV/erysipelas vaccination of sows by cycle, as well ADV vaccination in a blanket fashion. Gilts were vaccinated against PRRSV and PPV/erysipelas during the acclimation period. The performed study was approved by The Zoetis Olot Animal Welfare Committee prior to the start of the experiment, with reference number 382, and it was notified to and approved by Spanish Authorities.

From the 121 total sampled sows, 57 were primiparous and 64 were multiparous (≥ second parity) (Table 1). All dams, primiparous and multiparous, were sampled at two time points (S1 and S2). Additionally, tissues (brain and lung) from a total of 255 mummified or stillborn piglets from the respective sampled sows were also included in this study, except for primiparous ones from farm B, from which the fetuses were not available for the study (Table 2). The number of collected fetuses ranged from 1 to 18 animals per litter. The time of gestation at which the fetuses died was determined by its body size at delivery and physical aspect [31]. Thus, by the physical aspect, the fetuses were classified as mummified (*n* = 49) or stillborn (*n* = 206) [32]. Noteworthy, all fetuses (mummified and stillborn) were collected at the expected farrowing time.

### 4.2. DNA Extraction, PCR and qPCR

Tissue samples from the fetuses (brain and lungs) were homogenized separately. DNA extraction from 200 μL of macerated tissue supernatant as well as 200 μL of sera from sows was performed using a MagMAx™ Pathogen RNA/DNA Kit (Applied Biosystems^®^) according to the manufacturer’s protocol. 

A conventional PCR targeting the PCV-3 rep gene region (ORF1) was performed as previously described [8]. The PCR products were checked by electrophoresis on 1.5% TAE agarose gel. 

To quantify the amount of virus in the PCR positive samples, a real-time quantitative PCR (qPCR) was performed also as previously described [8,33]. The qPCR results were expressed in log_10_ of PCV-3 DNA copies/µL of serum or supernatant of macerated tissues sample.

### 4.3. PCV-3 Phylogenetic Analysis

Those qPCR positive samples with the highest amount of virus in both fetal tissue supernatant (from 2.74 to 7.84 log_10_ copies/µL) and sow serum samples (from 0.88 to 1.91 log_10_ copies/µL) were selected to be sequenced by means of PCRs amplifying the whole PCV-3 genome [17]. When possible, samples from sows and fetuses from the same litter (with a high amount of virus) were selected. The PCR reaction contained 1 × PCR buffer, 0.4 µM of dNTPs, 0.2 µM of each primer, 1 Unit of DNA polymerase Platinum™ SuperFi™ (Invitrogen™), and water to bring the final volume up to 50 μL. The thermal conditions included 98 °C for 5 min followed by 40 cycles of 98 °C for 30 s, 55 °C for 1 min and 72 °C for 2 min, plus the final elongation at 72 °C for 7 min. The obtained amplicons were purified with an ExoSA*P*-IT^®^ Express PCR product Cleanup (Thermo Fisher Scientific) Kit according to the manufacturer’s protocol and sequenced by the Sanger method (ABI 3730XL - Macrogen Europe, Madrid, Spain). The quality of the sequences was analyzed by the Finch TV software and trimmed in BioEdit vs. 7.2.6 [34]. Obtained amplicons were assembled using the reference mapped-based strategy [35] to achieve PCV-3 complete genomes. The Integrative Genomics Viewer software [36] was used for visualizing the assembly and extracting the consensus sequence. The complete genome sequences were aligned in BioEdit vs. 7.2.6 [35] with summarized collected reference samples according to a previously published method [21], as well as the ORF2 gene and the translated ORF2 region with the standard genetic code (using MEGAX). The best substitution method was selected based on the lowest Bayesian Information Criterion (BIC) score calculated on MEGAX software [37], either for the complete genome analysis as for the ORF2 aa analysis. The Maximum Likelihood tree with Hasegawa–Kishino–Yano (HKY) model [38] plus Gamma distribution phylogenetic was used to construct the phylogenetic tree for the complete genome and the tree for the ORF2 gene both with 1000 bootstrap replicates using MEGAX software [37]. The translated ORF2 region was used to build a Neighbor Joining (NJ) phylogenetic tree constructed using the Jones–Taylor–Thornton’s model [39] with 1000 bootstraps. The identity among nucleotide sequences was compared using Clustal Omega [40].

All PCV-3 sequences generated in this study were deposited at the NCBI GenBank with the accession numbers MT350517–MT350555.

### 4.4. Statistical Analysis

The frequency of PCV-3 DNA detection in serum samples from primiparous and multiparous sows was compared globally, per farm (only from primiparous) and per sampling point. The frequency of detection as well as the median PCV-3 viral load in fetal samples were analyzed considering the type of tissue (lung or brain), the type of fetus (mummies or stillborn), their dam (primiparous or multiparous), and the farm of origin. The frequencies of detection were analyzed using the Pearson’s Chi-squared test or Fisher´s exact test. The median PCV-3 viral loads were compared using the Mann-Whitney test. These analyses were carried out using GraphPad software (GraphPad software Inc.) and GraphPad Prism 8, where *P*-value < 0.05 was considered statistically significant. 

## Figures and Tables

**Table 1:** Number of Porcine circovirus 3 (PCV-3) PCR positive serum samples out of the total number of tested samples (percentage) per farm and sampling point in primiparous and multiparous sows.

Farm	Sampling point	Primiparous Sows	Multiparous Sows	Total
A	S1	1/19 (5.3%)	0/25 (0.0%)	1/44 (2.3%)
	S2	8/19 (42.1%)	0/25 (0.0%)	8/44 (18.2%)
B	S1	0/17 (0.0%)	0/20 (0.0%)	0/37 (0.0%)
	S2	3/17 (17.6%)	0/20 (0.0%)	3/37 (8.1%)
C	S1	0/21 (0.0%)	0/19 (0.0%)	0/40 (0.0%)
	S2	7/21 (33.3%)	0/19 (0.0%)	7/40 (17.5%)

Eighty-six out of 255 (33.7%) fetuses had at least one tissue positive for PCV-3 genome detection (Table 2), being 41 out of 96 (42.7%) from farm A, 2 out of 48 (4.2%) from farm B, and 43 out of 111 (38.7%) from farm C. The numbers of positive fetuses from farms A and C were not statistically different between them (*P*-value = 0.6613), but values from farms A and C were statistically different from those of farm B (*P*-value < 0.0001 in both cases). These 86 fetuses positive to PCV-3 PCR came from 14 positive (all primiparous) and 31 negative dams (being 23 primiparous and 8 multiparous sows).

**Table 2:** Number and percentage of mummified fetuses and stillborn with at least one PCV-3 PCR-positive tissue and their viral load range.

Farm	Sow Parity	Mummified Fetuses		Stillborn		Total
		PCR Positive/Total (%)	Viral Load (Min-Max) log_10_ PCV-3 Copies/µL	PCR Positive/Total (%)	Viral Load (Min-Max) log_10_ PCV-3 Copies/µL	
A	Primiparous	13/15 (86.7%)	0.66–6.48	19/26 (73.1%)	0.56–6.66	32/41 (78.0%) ^A^
	Multiparous	0/0 (0.0%)	-	9/55 (16.4%)	0.15–1.96	9/55 (16.4%) ^B^
B *	Multiparous	0/10 (0.0%)	-	2/38 (5.3%)	1.67–2.26	2/48 (4.2%)
C	Primiparous	13/15 (86.7%)	0.94–7.84	28/35 (80.0%)	0.30–6.46	41/50 (82.0%) ^A^
	Multiparous	1/9 (11.1%)	1.01	1/52 (1.9%)	0.56	2/61 (3.3%) ^B^
Total		27/49 (55.1%) ^a^	0.66–7.84	59/206 (28.6%)^b^	0.15–6.66	86/255 (33.7%)

* No fetuses from primiparous sows were available from farm B. Different letters in superscript in a row mean statistically significant differences between the total of mummies and stillborn (*P*-value < 0.05). Different letters different letters in upper case in a column mean statistically significant differences between fetus from primiparous and multiparous sows (*P*-value < 0.05).

**Table 3:** Number and percentage of PCV-3 PCR positive tissues from mummies or stillborn.

Farm	Fetuses	Tissue		Total Tissue PCR Results (%)
		Brain PCR Results (%)	Lung PCR Results (%)	
A	Mummies	10/15 (66.7%) ^a^	13/15 (86.7%) ^a^	23/30 (76.7%) ^a^
	Stillborn	24/81 (29.6%) ^b^	25/81 (30.9%) ^b^	49/162 (30.2%) ^b^
B	Mummies	0/10 (0.0%)	0/10 (0.0%)	0/20 (0.0%)
	Stillborn	0/38 (0.0%)	2/38 (5.3%)	2/76 (2.6%)
C	Mummies	11/24 (45.8%) ^a^	14/24 (58.3%) ^a^	25/48 (52.1%) ^a^
	Stillborn	21/87 (24.1%) ^b^	27/87 (31.0%) ^b^	48/174 (27.6%) ^b^
	Total	66/255 (25.9%)	81/255 (31.8%)	147/510 (28.8%)

Different letters in superscript in a column mean statistically significant differences in PCV-3 detection between mummies and stillborn in each farm (*P*-value < 0.05).

## Supplementary Materials

The following supplementary material is available online at https://www.mdpi.com/2076-0817/9/7/533/s1, Figure S1. PCV-3 phylogenetic ML tree constructed with ORF2 gene using the Hasegawa–Kishino–Yano (HKY) model with Gamma distribution at 1000 bootstrap (the width of the branches is proportional to bootstrap *P*-values) and with GenBank reference samples included in Franzo et al., 2020. The 39 PCV-3 ORF2 sequence samples were labeled with GenBank ID followed by farm identification, sow number, and sample number; F, for fetus samples; B, for brain; or L for lung tissue. Samples were colored by farm (farm A in red, farm B in blue, and farm C in green). Figure S2. Phylogenetic NJ tree of aa of CAP gene constructed using Jones–Taylor–Thornton’s model at 1000 bootstrap (the width of the branches is proportional to bootstrap *P*-values) and with GenBank reference samples included in Franzo et al., 2020. The 39 PCV-3 ORF2 aa sequences were labeled with GenBank ID followed by farm identification, sow number, and sample number; F, for fetus samples; B, for brain; or L for lung tissue. Samples were colored by farm (farm A in red, farm B in blue, and farm C in green.
