# Supplementary figures and images for: Frequency of Detection and Phylogenetic Analysis of Porcine circovirus 3 (PCV-3) in Healthy Primiparous and Multiparous Sows and Their Mummified Fetuses and Stillborn
Source: Pathogens. 2020 Jul 2;9(7):533. doi: 10.3390/pathogens9070533 (PMC7399965; doi:10.3390/pathogens9070533)

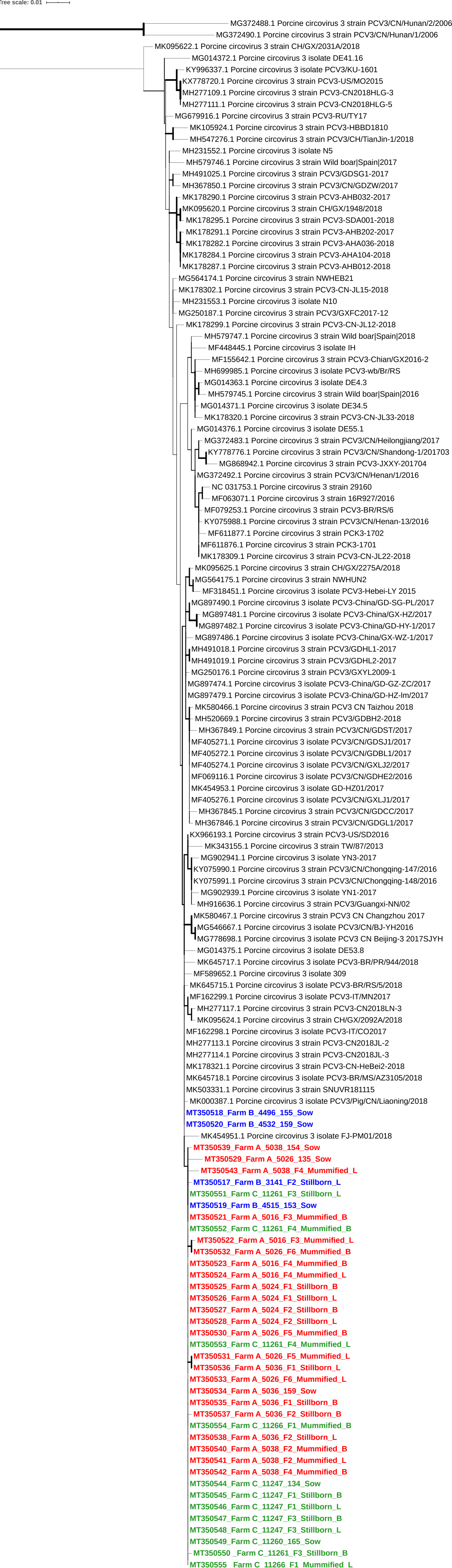

Tree scale: 0.01

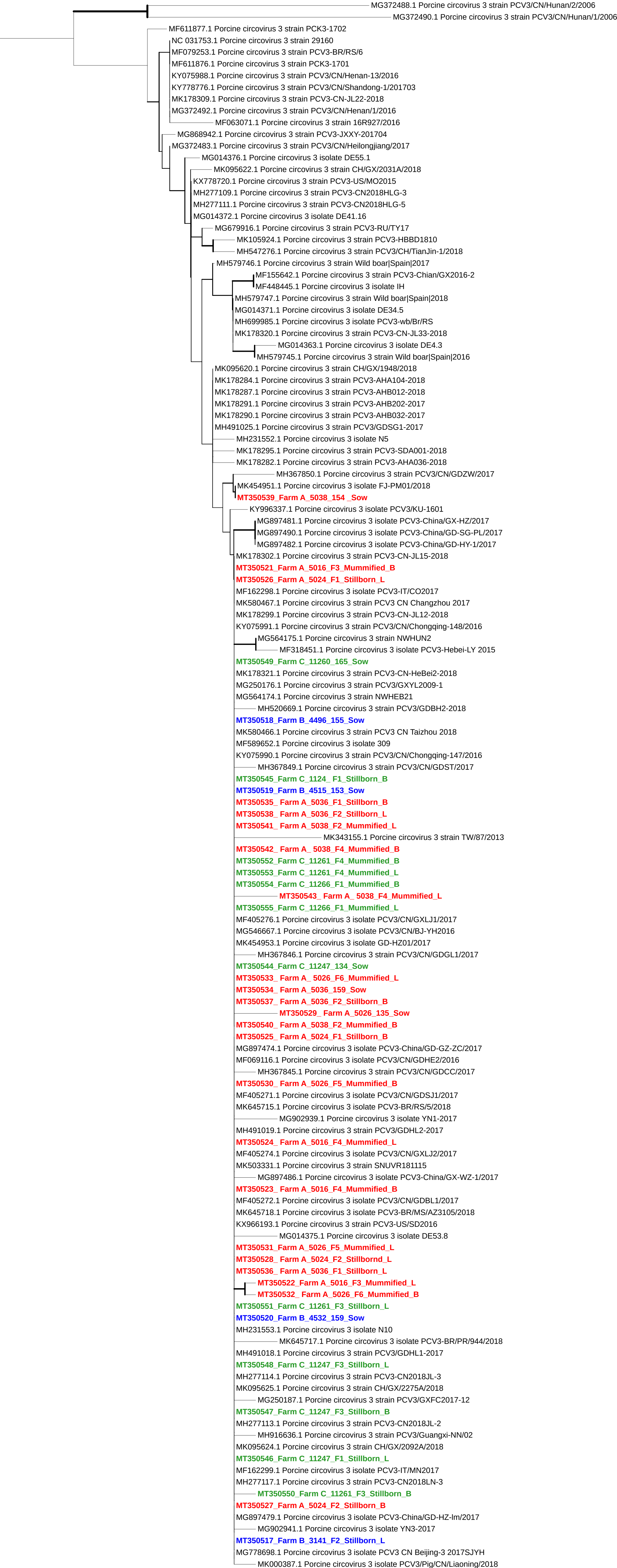

Supplement: Supplementary file 1 [file pathogens-09-00533-s001.pdf]
